# Supplementary material for: Quantification and Optimization of Ethanolic Extract Containing the Bioactive Flavonoids from Millettia pulchra Radix
Source: Molecules. 2021 Jun 15;26(12):3641. doi: 10.3390/molecules26123641 (PMC8232251; doi:10.3390/molecules26123641)
Supplement: Supplementary file 1 [file molecules-26-03641-s001.zip › molecules-1247115-SI.pdf]

Quantification and Optimization of Ethanolic Extract Containing the Bioactive Flavonoids from *Millettia pulchra* radix

List of Supplementary Material

Table S1. The ANOVA results for the response surface quadratic models for the yield from *M. pulchra* radix.
Table S2. The ANOVA results for the response surface quadratic models for the content of **1** from *M. pulchra* radix.
Table S3. The ANOVA results for the response surface quadratic models for the content of **2** from *M. pulchra* radix.
Table S4. The ANOVA results for the response surface quadratic models for the content of **3** from *M. pulchra* radix.
Table S5. The ANOVA results for the response surface quadratic models for the content of **4** from *M. pulchra* radix.
Figure S1. The ^{1}H NMR spectrum of compound **1** (500 MHz in chloroform-*d*)
Figure S2. The ^{13}C NMR spectrum of compound **1** (125 MHz in chloroform-*d*)
Figure S3. The ^{1}H NMR spectrum of compound **2** (500 MHz in chloroform-*d*)
Figure S4. The ^{13}C NMR spectrum of compound **2** (125 MHz in chloroform-*d*)
Figure S5. The ^{1}H NMR spectrum of compound **3** (500 MHz in chloroform-*d*)
Figure S6. The ^{13}C NMR spectrum of compound **3** (125 MHz in chloroform-*d*)
Figure S7. The ^{1}H NMR spectrum of compound **4** (500 MHz in chloroform-*d*)
Figure S8. The ^{13}C NMR spectrum of compound **4** (125 MHz in chloroform-*d*)

Table S1. The ANOVA results for the response surface quadratic models for the yield from *M. pulchra* radix.

Source (Y_1)	Sum of squares (SS)	df	Mean square	F-value	p-value
Model	1225.16	9	141.5	57.05	< 0.0001
X_1	445.94	1	499.69	201.46	< 0.0001
X_2	54.11	1	51.98	20.96	0.001
X_3	393.34	1	393.72	158.73	< 0.0001
X_1X_2	27	1	27.91	11.25	0.0073
X_1X_3	40.97	1	41.48	16.72	0.0022
X_2X_3	25.52	1	25.65	10.34	0.0092
X_1^2	76.86	1	68.62	27.67	0.0004
X_2^2	14.02	1	8.79	3.54	0.0892
X_3^2	38.69	1	41.7	16.81	0.0021
Residual	23.11	10	2.48		
Lack of fit	22.97	6	4.12	262.15	< 0.0001
Pure error	0.1367	4	0.0157		
Core total	1248.27	19			
R^2	0.9815		PRESS	249.3	
R^2_{adj}	0.9648		CV%	8.74	
R^2_{pre}	0.8003		AP	29.96	

Y_1: Extraction yield (%)

X_1: Extraction time; X_2: Ratio of solvent/material; X_3: Concentration of ethanol (%)

Table S2. The ANOVA results for the response surface quadratic models for the content of **1** from *M. pulchra* radix.

Source (Y_2)	Sum of squares (SS)	df	Mean square	F-value	p-value
Model	500.99	9	55.67	128.06	< 0.0001
X_1	13.05	1	13.05	30.03	0.0003
X_2	0.4983	1	0.4983	1.15	0.3095
X_3	391.06	1	391.06	899.68	< 0.0001
X_1X_2	0.2817	1	0.2817	0.6481	0.4395
X_1X_3	6.37	1	6.37	14.65	0.0033
X_2X_3	1.64	1	1.64	3.78	0.0805
X_1^2	0.0745	1	0.0745	0.1714	0.6876
X_2^2	0.6187	1	0.6187	1.42	0.2604
X_3^2	50.95	1	50.95	117.22	< 0.0001
Residual	4.35	10	0.4347		
Lack of fit	4.28	6	0.7141	45.92	0.0012
Pure error	0.0622	4	0.0155		
Core total	505.33	19			
R^2	0.9914		PRESS	31.85	
R^2_{adj}	0.9837		CV%	10.40	
R^2_{pre}	0.937		AP	31.87	

Y_2: Content of compound **1** (μg/g)

X_1: Extraction time; X_2: Ratio of solvent/material; X_3: Concentration of ethanol (%)

Table S3. The ANOVA results for the response surface quadratic models for the content of **2** from *M. pulchra* radix

Source (Y_3)	Sum of squares (SS)	df	Mean square	F-value	p-value
Model	6203.21	9	689.25	98.26	< 0.0001
X_1	455.29	1	455.29	64.91	< 0.0001
X_2	374.02	1	374.02	53.32	< 0.0001
X_3	5166.01	1	5166.01	736.49	< 0.0001
X_1X_2	16.85	1	16.85	2.4	0.1522
X_1X_3	16.16	1	16.16	2.3	0.16
X_2X_3	91.4	1	91.4	13.03	0.0048
X_1^2	68.71	1	68.71	9.79	0.0107
X_2^2	12.98	1	12.98	1.85	0.2036
X_3^2	3.44	1	3.44	0.4901	0.4999
Residual	70.14	10	7.01		
Lack of fit	68.67	6	11.45	31.12	0.0026
Pure error	1.47	4	0.3677		
Core total	6273.35	19			
R^2	0.9888		PRESS	435.54	
R^2_{adj}	0.9788		CV%	5.69	
R^2_{pre}	0.9306		AP	36.57	

Y_3: Content of compound **2** (μg/g)

X_1: Extraction time; X_2: Ratio of solvent/material; X_3: Concentration of ethanol (%)

Table S4. The ANOVA results for the response surface quadratic models for the content of **3** from *M. pulchra* radix

Source (Y_4)	Sum of squares (SS)	df	Mean square	F-value	p-value
Model	132.36	9	14.71	97.93	< 0.0001
X_1	8.02	1	8.02	53.41	< 0.0001
X_2	0.7165	1	0.7165	4.77	0.0539
X_3	103.7	1	103.7	690.5	< 0.0001
X_1X_2	0.0255	1	0.0255	0.1699	0.6889
X_1X_3	1.11	1	1.11	7.36	0.0218
X_2X_3	0.0915	1	0.0915	0.6095	0.4531
X_1^2	2.09	1	2.09	13.89	0.0039
X_2^2	0.324	1	0.324	2.16	0.1726
X_3^2	14.54	1	14.54	96.8	< 0.0001
Residual	1.5	10	0.1502		
Lack of fit	1.33	6	0.222	5.23	0.0656
Pure error	0.1697	4	0.0424		
Core total	133.86	19			
R^2	0.9888		PRESS	7.99	
R^2_{adj}	0.9787		CV%	11.02	
R^2_{pre}	0.9403		AP	30.91	

Y_4: Content of compound **3** (μg/g)

X_1: Extraction time; X_2: Ratio of solvent/material; X_3: Concentration of ethanol (%)

Table S5. The ANOVA results for the response surface quadratic models for the content of **4** from *M. pulchra* radix

Source (Y_5)	Sum of squares (SS)	df	Mean square	F-value	p-value
Model	42.12	9	4.68	32.53	< 0.0001
X_1	2.2	1	2.2	15.26	0.0029
X_2	1.25	1	1.25	8.68	0.0146
X_3	26.04	1	26.04	181	< 0.0001
X_1X_2	0.8378	1	0.8378	5.82	0.0365
X_1X_3	1.96	1	1.96	13.61	0.0042
X_2X_3	0.7556	1	0.7556	5.25	0.0449
X_1^2	0.0027	1	0.0027	0.0187	0.8941
X_2^2	5.35E-06	1	5.35E-06	0	0.9953
X_3^2	5.94	1	5.94	41.29	< 0.0001
Residual	1.44	10	0.1439		
Lack of fit	1.43	6	0.2382	100.12	0.0003
Pure error	0.0095	4	0.0024		
Core total	43.56	19			
R^2	0.967		PRESS	21.51	
R^2_{adj}	0.9372		CV%	19.36	
R^2_{pre}	0.5061		AP	20.09	

Y_5: Content of compound **4** (μg/g)

X_1: Extraction time; X_2: Ratio of solvent/material; X_3: Concentration of ethanol (%)

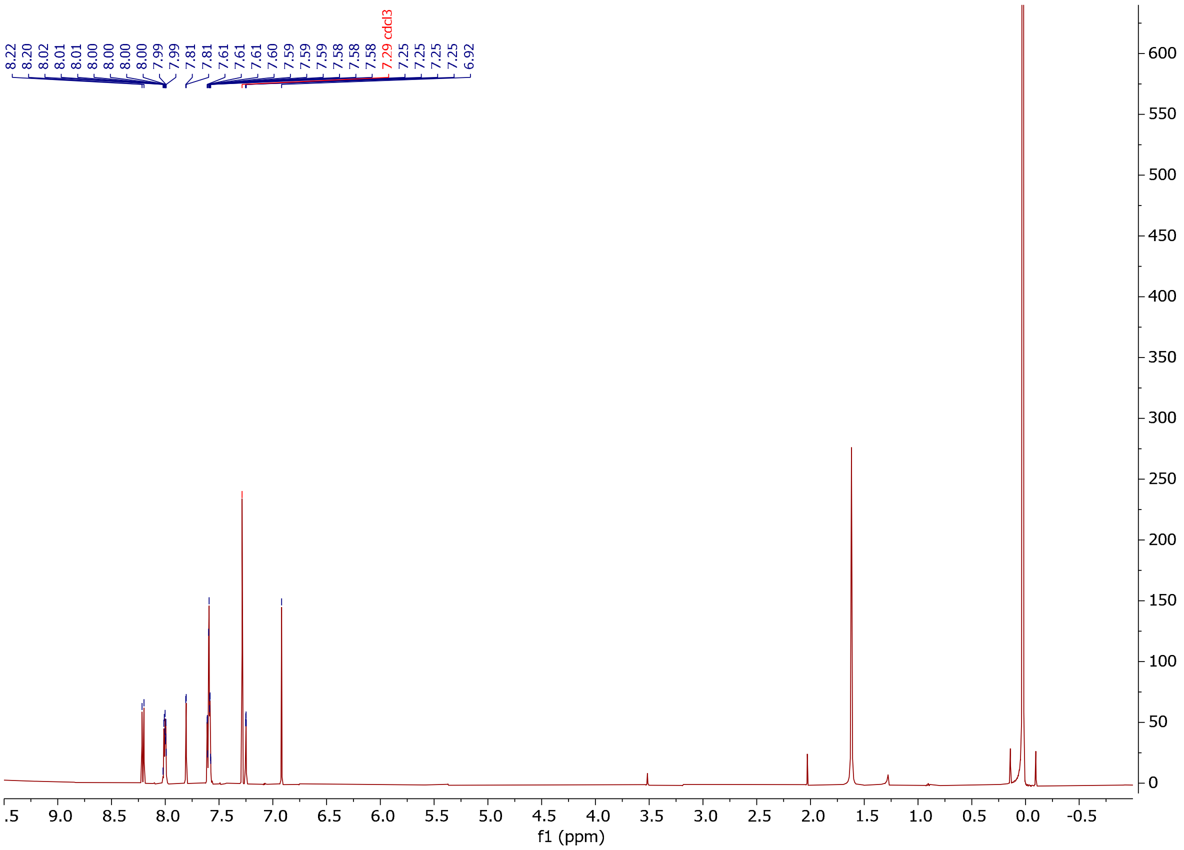

Figure S1. The ¹H NMR spectrum of compound **1** (500 MHz in chloroform-*d*)

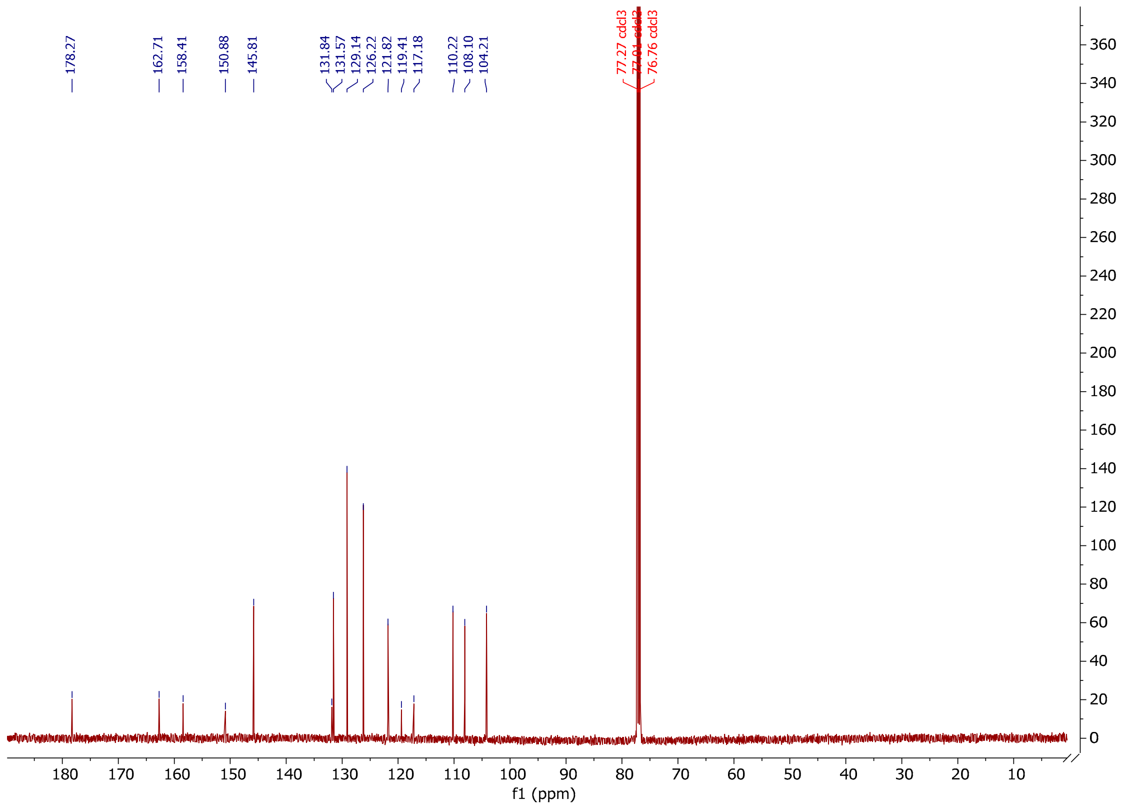

Figure S2. The ^{13}C NMR spectrum of compound **1** (125 MHz in chloroform-*d*)

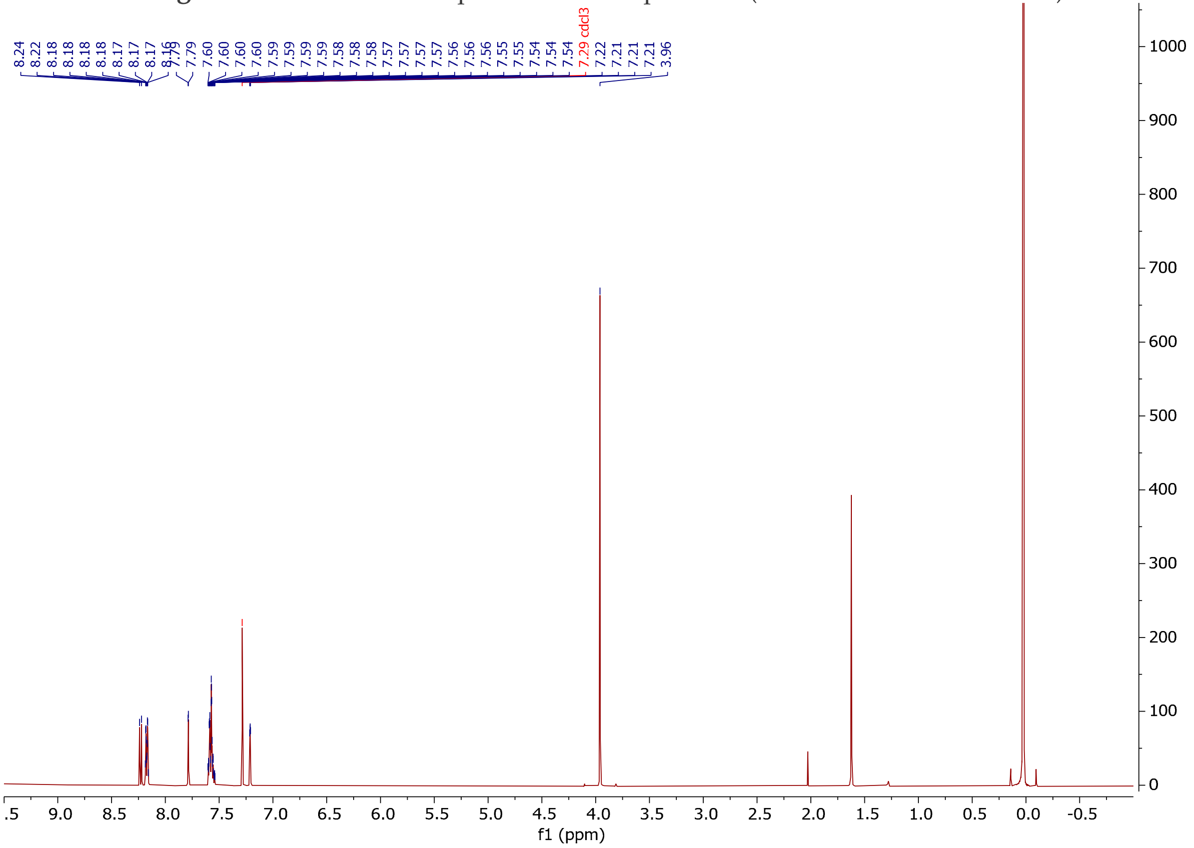

Figure S3. The ^{1}H NMR spectrum of compound **2** (500 MHz in chloroform-*d*)

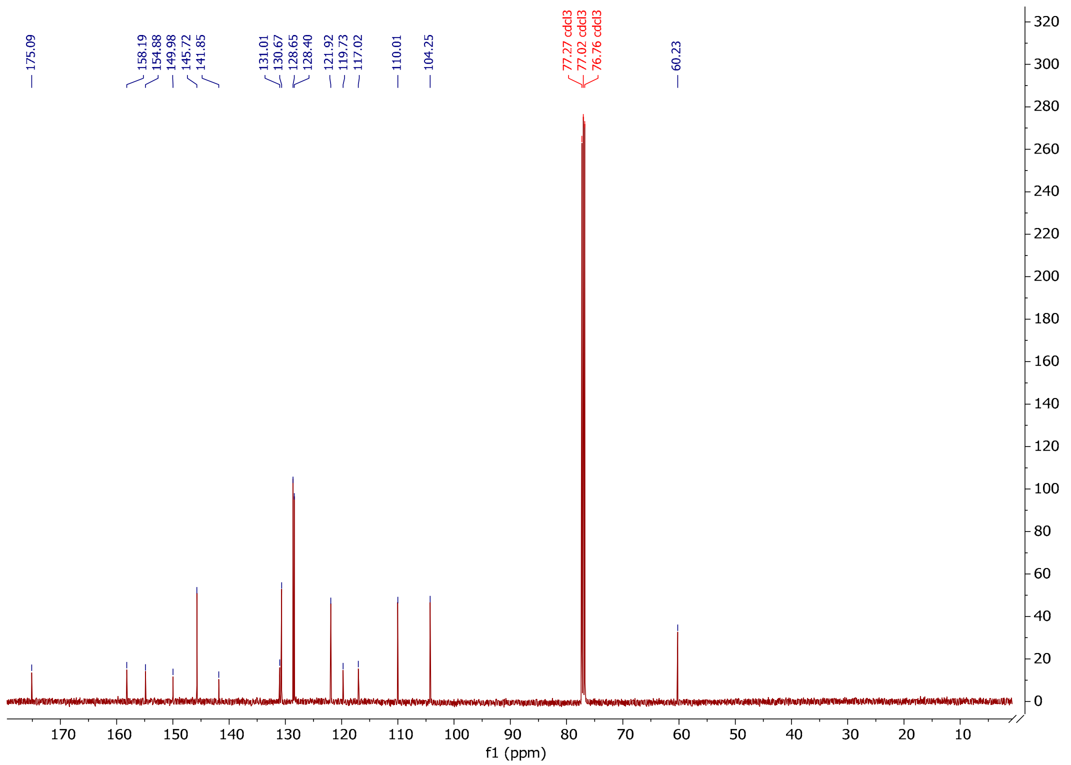

Figure S4. The [1]C NMR spectrum of compound **2** (125 MHz in chloroform-*d*)

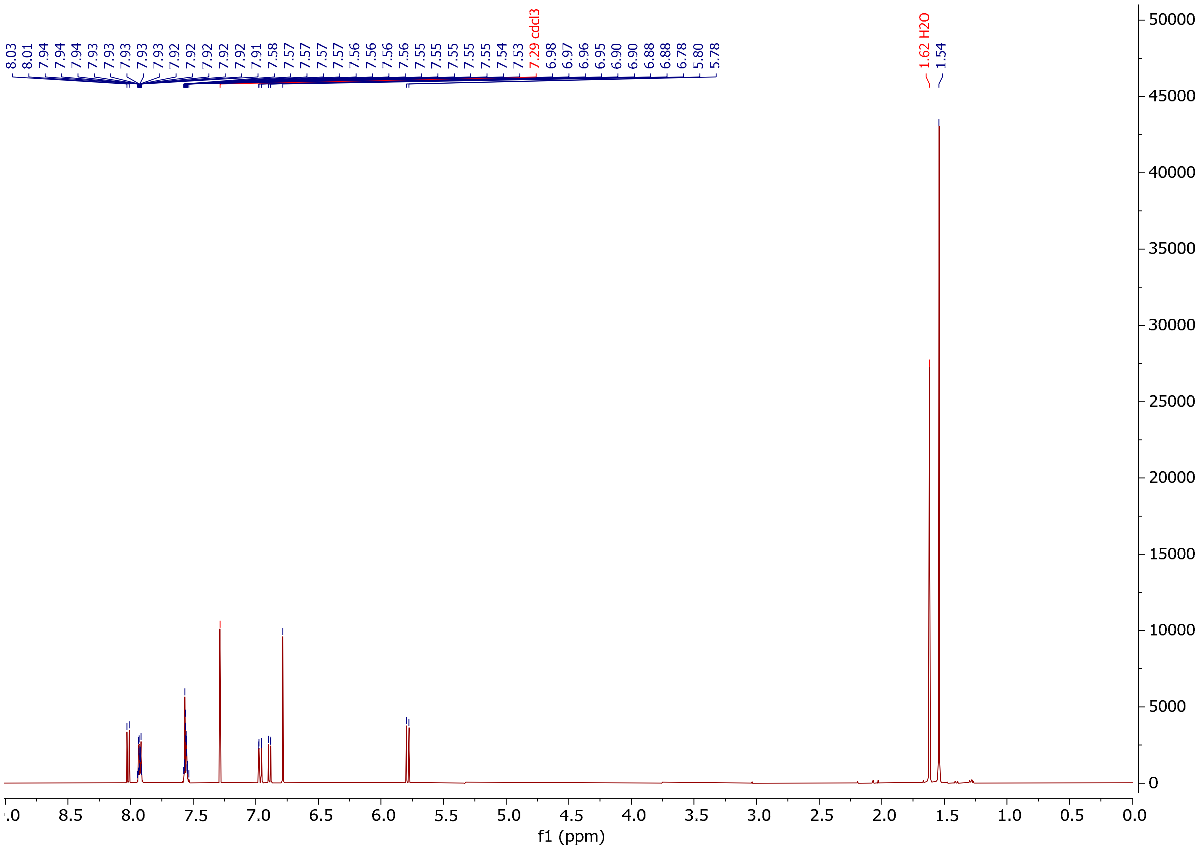

Figure S5. The ^{1}H NMR spectrum of compound **3** (500 MHz in chloroform-*d*)

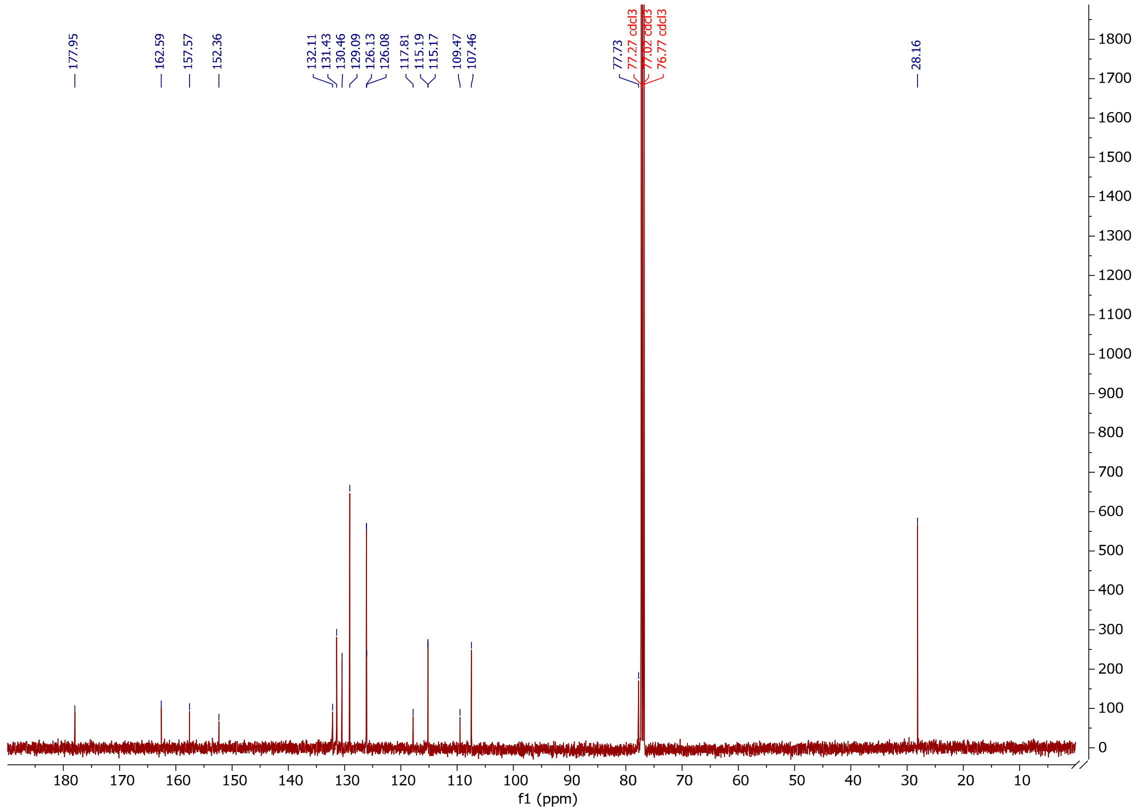

Figure S6. The ^{13}C NMR spectrum of compound **3** (125 MHz in chloroform-*d*)

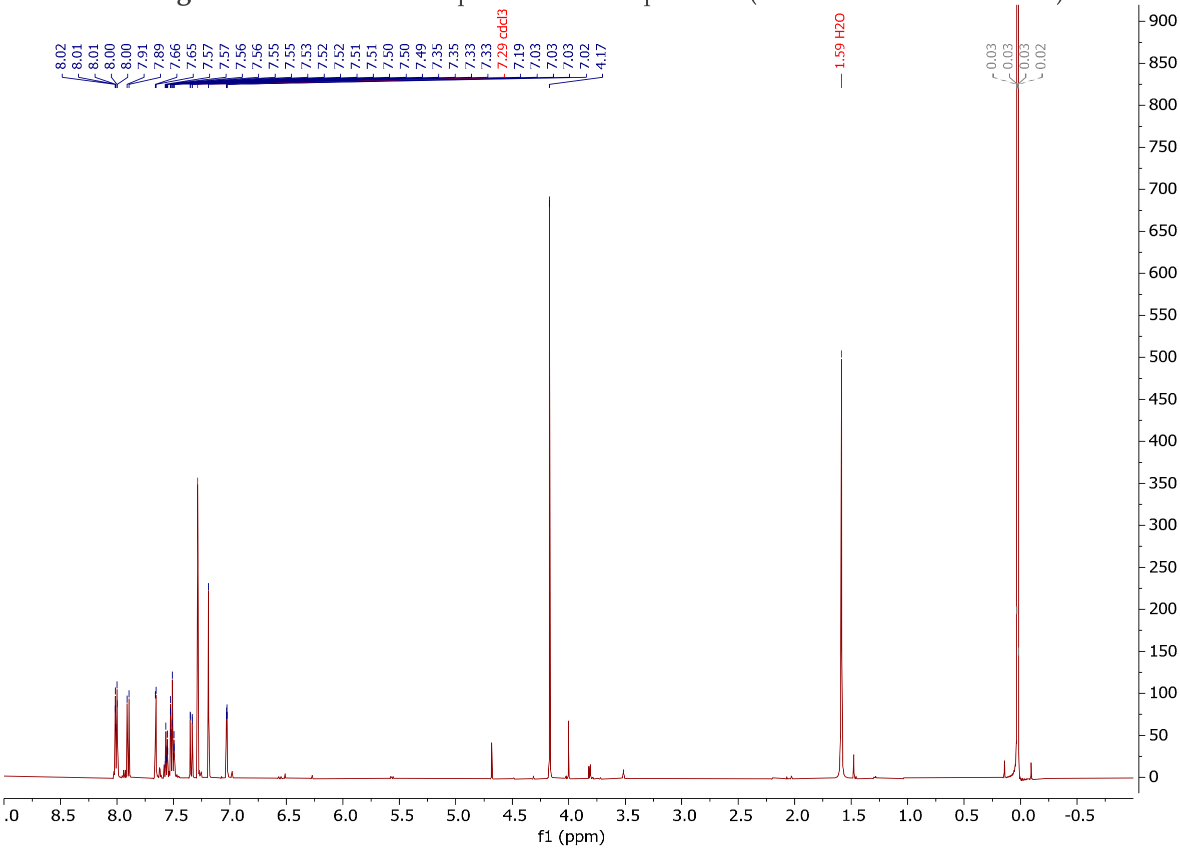

Figure S7. The ^{1}H NMR spectrum of compound **4** (500 MHz in chloroform-*d*)

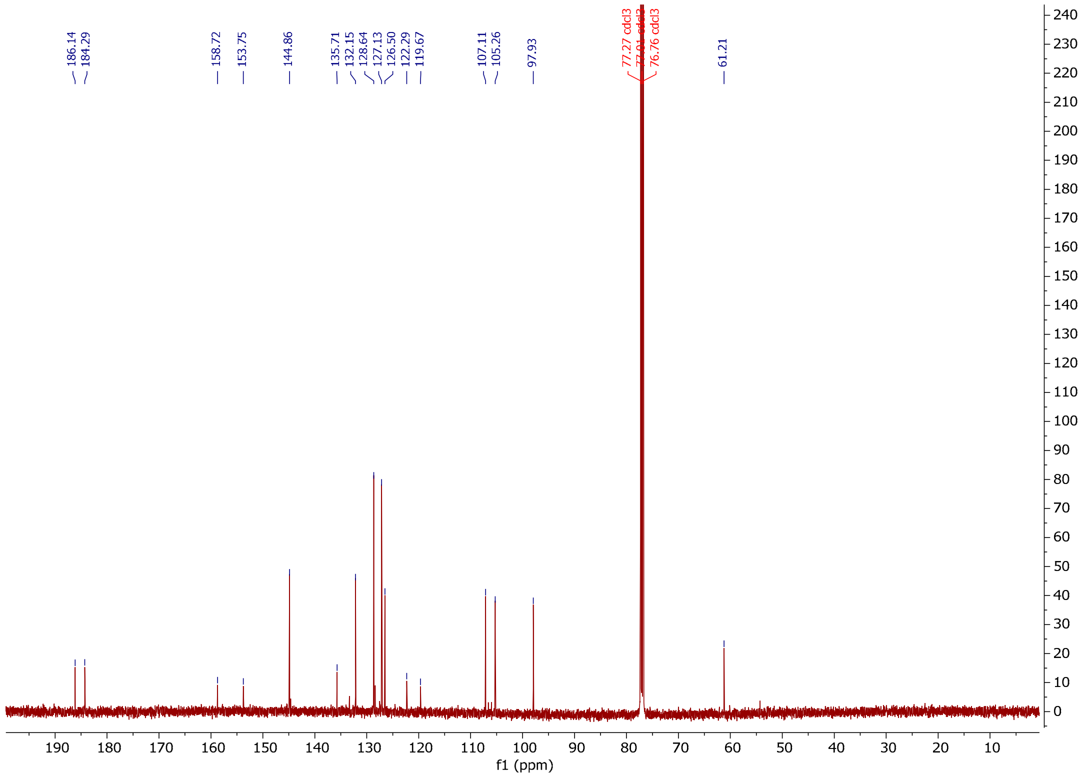

Figure S8. The ^{13}C NMR spectrum of compound **4** (125 MHz in chloroform-*d*)
